# Life cycle cost and environmental assessment for resource-oriented toilet systems

**DOI:** 10.1016/j.jclepro.2018.06.129

**Published:** 2018-09-20

**Authors:** Yilei Shi, Lu Zhou, Yangyu Xu, Hongjie Zhou, Lei Shi

**Affiliations:** School of Environment, Tsinghua University, Beijing, 100084, PR China

**Keywords:** Toilet, Resource recovery, Forward osmosis, Cost-benefit analysis, Life cycle assessment, SA, Scenario A, SB_1_, Scenario B_1_, SB_2_, Scenario B_2_, SC_1_, Scenario C_1_, SC_2_, Scenario C_2_, SC_3_, Scenario C_3_, SC_4_, Scenario C_4_, FO, forward osmosis, RO, reverse osmosis, ED, electrodialysis, LCA, life cycle assessment, ENPV, net economic present value, CNY, China Yuan, USD, United States dollar, TOrCs, trace organic compounds, STPs, sewage treatment plants, N, nitrogen, NH_3_-N, ammonia nitrogen, TN, total nitrogen, P, phosphorus, TP, total phosphorus, COD_Cr_, dichromate oxidizability, K, potassium, TDS, total dissolved solids, GWP, Global Warming Potential, AP, Acidification Potential, EP, Eutrophication Potential, ODP, Ozone Layer Depletion Potential, ADP elements, Abiotic Depletion Elements, ADP fossil, Abiotic Depletion Fossil, FAETP, Freshwater Aquatic Ecotoxicity, HTP, Human Toxicity Potential, MAETP, Marine Aquatic Ecotoxity, POCP, Photochem. Ozone Creation Potential, TETP, Terrestric Ecotoxicity Potential (TETP), R11, trichlorofluoromethane, Sb, antimony, DCB, 4,4′-diaminobiphenyl

## Abstract

The rich content of nutrients in human waste provides an outlook for turning it from pollutants to potential resources. The pilot-scale resource-oriented toilet with forward osmosis technology was demonstrated to have advantages to recover clean water, nitrogen, phosphorus, potassium, biogas, and heat from urine and feces. For the possibility of further full-scale implementation in different scenarios, six resource-oriented toilet systems and one conventional toilet system were designed in this study. The methodology of cost-benefit analysis and life cycle assessment were applied to analyze the life cycle economic feasibility and environmental sustainability of these systems. As results indicated, resource-oriented toilets with forward osmosis technology concentrating urine proved to have both economic and environmental benefit. The economic net present value results of new resource-oriented toilets were much better than conventional toilet. The energy consumption in resource-oriented toilets contributes a lot to the environmental impacts while resource recovery such as the fertilizer production and fresh water harvest in resource-oriented toilet systems offsets a lot. Taking both life cycle economic feasibility and environmental sustainability into consideration, the partial resource-oriented toilet (only recovering nutrients from urine) is the best choice, and the totally independent resource-oriented toilet could be applied to replace conventional toilets in areas without any external facilities such as sewer and water supply system *etc*.

## Highlights

• The forward osmosis technology was applied in resource-oriented toilets.
• Cost-benefit analysis and life cycle assessment were conducted on resource-oriented toilets.
• Resource-oriented toilets have higher economic net present values and lower environmental cost.
• The resource-oriented toilets have better economic feasibility and environmental sustainability.

## Graphical abstract

**Figure:**
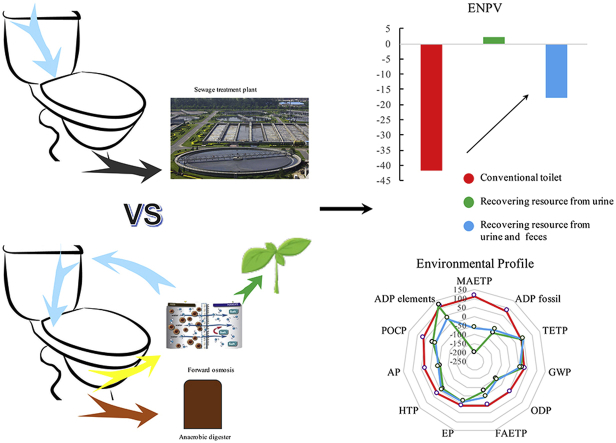

## 1 Introduction

The discharge of wastewater from the toilet containing human waste and flush water will not only cause the waste of nutrients and clean water, but also increase the difficulties of the sewage treatment. Averagely, an adult produces 1.5 kg urine and 0.14 kg feces per day, containing 11.5 g N, 1.5 g P and 3.15 g K, and of which about 88% of the N, 67% of the P and 73% of the K are contained in the urine (Benetto et al., 2009; Karak and Bhattacharyya, 2011). The urine contributes about 80% nitrogen, 50% phosphorus and 90% potassium in domestic wastewater (Larsen et al., 2001; Vinnerås and Jönsson, 2002). A typical secondary sewage treatment plant consumes 0.3–0.6 kWh electricity in treating 1 m^3^ wastewater per day (McCarty et al., 2011). Additionally, the energy consumption in conveyance is several times more than that in treatment (Englehardt et al., 2013). The massive energy consumption in collection and treatment will finally result in negative impacts of environment, waste of resource and cost in construction and operation (Yan et al., 2017). Therefore, there is a paradox in existing sewage treatment processes, that is, the use of a large number of energy and chemicals to treat sewage, but eventually results in a huge waste of resource and a heavy burden to both environment and economy.

In the face of these problems, recovering resource and energy from human waste becomes a global consensus (Larsen et al., 2016; Trimmer et al., 2017). A lot of new treatment processes have been developed. For example, the struvite precipitation was applied to recover nitrogen and phosphorus (Lind et al., 2000; Etter et al., 2011; de Boer et al., 2018); the electrochemical treatment processes were used to recovering nitrogen and disinfection (Cho and Hoffmann, 2014; Huang et al., 2016; Ledezma et al., 2017); the combustion was used to process human waste into burnable fuel (Stokes et al., 2014); the hydrothermal carbonization technology was used to reuse human biowastes as some kinds of safe material and recover nutrients at the same time (Afolabi et al., 2014); the dehydration was applied for volume reduction of the urine (Senecal and Vinneras, 2017); the adsorption technology was used to ensure the safety of recovered products (Simha and Ganesapillai, 2017); the membrane technology was used to concentrate the urine (Künzle et al., 2015). Besides, there are even some hybrid technologies, such as the hybrid process of membrane-based pre-concentration and ion exchange for recovering clean water and nitrogen (Gong et al., 2017), the hybrid of flocculation and nutrient precipitation for recovering nutrients from urine (Wang et al., 2018), *etc*. The low recovered nutrient concentration or complicated processes, however, limit the further development and practical application of these technologies.

As a sustainable membrane technology, forward osmosis has a significant advantage in the concentration of nutrient-rich wastewater. In FO system, there are a draw solution side running a high concentration solution and a feed solution side running the target solution that needs to be concentrated. In the concentration process, water diffuses from the feed solution into a draw solution through a dense, semipermeable FO membrane and the osmotic pressure is the driving force of water transport (Hancock et al., 2011; Lutchmiah et al., 2014). The main advantages of FO membrane technology in comparison with other membrane technologies such as reverse osmosis (RO), electrodialysis (ED) *etc.* are: *a*) no need for external pressure and low membrane strength requirement, *b*) low fouling propensity and quick recovery when polluted, *c*) high rejection to the ions (Cath et al., 2006). Based on the advantages of the FO, it enables concentration of a range of challenging, nutrient-rich streams, achieving high enrichment factors for streams (Xie et al., 2016). It can be used for the concentration of source-separated urine (yellow water) as well and the concentrated urine can be used as liquid fertilizer for agriculture and forestry. Thus, the aim of wastewater treatment and resource recovery will be both achieved.

At present, a number of researchers and institutions have been involved in this field. The FO membrane unit was used to concentrate the synthetic urine in a laboratory-scale (Zhang et al., 2014). National Aeronautics and Space Administration (NASA) used a two-stage FO infiltration system to treat urine and recycle water, and the rejection rate can reach more than 95%, and the recovery of water can reach 98% (Cath et al., 2005a; b). However, there were few practical sanitation application cases of the FO membrane technology. In view of this problem, a pilot-scale resource-oriented toilet serving 500 persons each day was built in the northwest corner of the playground of Tsinghua Primary School in October 2015 by our research team (Xu et al., 2017).

Simultaneously, it is also necessary to make a comprehensive assessment from economic and environmental perspective to determine the research priorities for the next step, evaluate the potential trade-offs for future expansion, and improve reliability before full-scale implementation. The main objective of this study is to design toilet systems for different scenarios based on the pilot-scale resource-oriented toilet using forward osmosis (FO) technology, and then evaluate economic feasibility and environmental sustainability of each system using the methodology of cost-benefit analysis and life cycle assessment.

## 2 Seven toilet systems

### 2.1 Performance of the resource-oriented toilet

In the actual operation of the toilet with FO units, the enrichment factor of yellow water was around 2.5. After enrichment, the enriched urine was used as liquid fertilizer for greening. The reclaimed water was used to flush toilets. Both FO and RO membrane had a high rejection rate for trace organic compounds (Hancock et al., 2011), ensuring safety of using reclaimed water to flush toilets. Additionally, the feces were digested to meet *Chinese Sanitary Standard for the Non-hazardous Treatment of Night Soil (GB7959-2012)*. The overall performance is shown in Table 1.

**Table 1:** The overall performance of the resource-oriented toilet system.

Items	Parameter	Before enrichment	After enrichment	Recovery rate
Urine	Volume (m^3^)	1.05 average	0.42 average	/
	pH	9.1–9.3	9.1–9.3	/
	TP (g/L)	2.11	4.14	78.48%
	TN (g/L)	2.46	3.16	51.38%
	NH_3_-N (g/L)	1.63	1.78	43.68%
	${\text{COD}}_{\text{Cr}}$ (g/L)	4.03	6.96	/
	K (g/L)	0.58	0.97	66.90%
	N + P + K (g/L)	4–6	9–11	/
Reclaimed water	Volume (m^3^)	/	0.63 average	60.00%
	TDS (mg/L)	/	800–1000	/

### 2.2 Designed toilet systems

Considering the differences of the water supply and drainage infrastructure conditions in different regions, in this study, seven toilet systems were designed, as shown in Table 2, to meet different potential requirements from pilot-scale to full-scale application promotion process.

**Table 2:** The necessary processes for different systems.

Scenarios	Water supply	Drainage	Yellow water treatment	Brown water treatment	Solar power b
Scenario A	√^a^	√	×	×	×
Scenario B_1_	√	√	√	×	√
Scenario B_2_	√	√	√	×	×
Scenario C_1_	√	×	√	√	√
Scenario C_2_	√	×	√	√	×
Scenario C_3_	×	×	√	√	√
Scenario C_4_	×	×	√	√	×

a √ = the scenario has this process, × = the scenario does not have this process.

b SB1, SC1, and SC3 are powered by photovoltaic cells while other scenarios are powered by the grid.

#### 2.2.1 Scenario A

Scenario A (SA) is a conventional public toilet system and there could be two operating modules applied in urban area and rural area respectively (Fig. A1). The system for urban area uses tap water to flush and discharges the wastewater into sewage treatment plants (STPs) finally. The system for rural area uses clean water to flush, then stores the wastewater in septic tanks, and eventually transports wastewater to STPs or directly reused after simple treatment.

#### 2.2.2 Scenario B_1_ and B_2_

Scenario B_1_ (SB_1_) and scenario B_2_ (SB_2_) are partial resource recovery toilet systems (Fig. A2). In these two scenarios, vacuum urine diversion toilets are used to get a source separate collection of urine (yellow water) and feces (brown water). Then, the FO system is used to concentrate the yellow water to obtain liquid fertilizer. A RO system is applied to recycle the draw solution used in FO system and recover flushing water from recycling process at the same time. The brown water is stored in the vacuum valve, and after a few days storage, the upper liquid in the vacuum valve would be discharged into sewer, and the sediment would be transported by trucks. Besides, SB_1_ relies on photovoltaic cells while SB_2_ is supported by electricity from power plants. These systems suit for areas with drainage facilities.

#### 2.2.3 Scenario C_1_, C_2_, C_3_ and C_4_

Scenario C_1_ (SC_1_), scenario C_2_ (SC_2_), scenario C_3_ (SC_3_) and scenario C_4_ (SC_4_) are the complete resource recovery toilet systems (Fig. A3). These systems treat brown water by anaerobic digestion on the basis of scenario B, becoming non-sewer systems. They can also be separated from the public water supply system when purify some surface water to produce enough flush water by RO system. Furthermore, this kind of system was divided into four scenarios (SC_1_, SC_2_, SC_3_ and SC_4_) depending on whether the water supply system is needed (SC_1_, SC_2_) or not (SC_3_, SC_4_) and what kind of power system is used, photovoltaic cells (SC_1_, SC_3_) or power plants (SC_2_, SC_4_). These systems suit for areas where sewage collection and treatment systems need a large number of investment and operating cost.

## 3 Data and methods

### 3.1 System boundaries

**Fig. 1:**
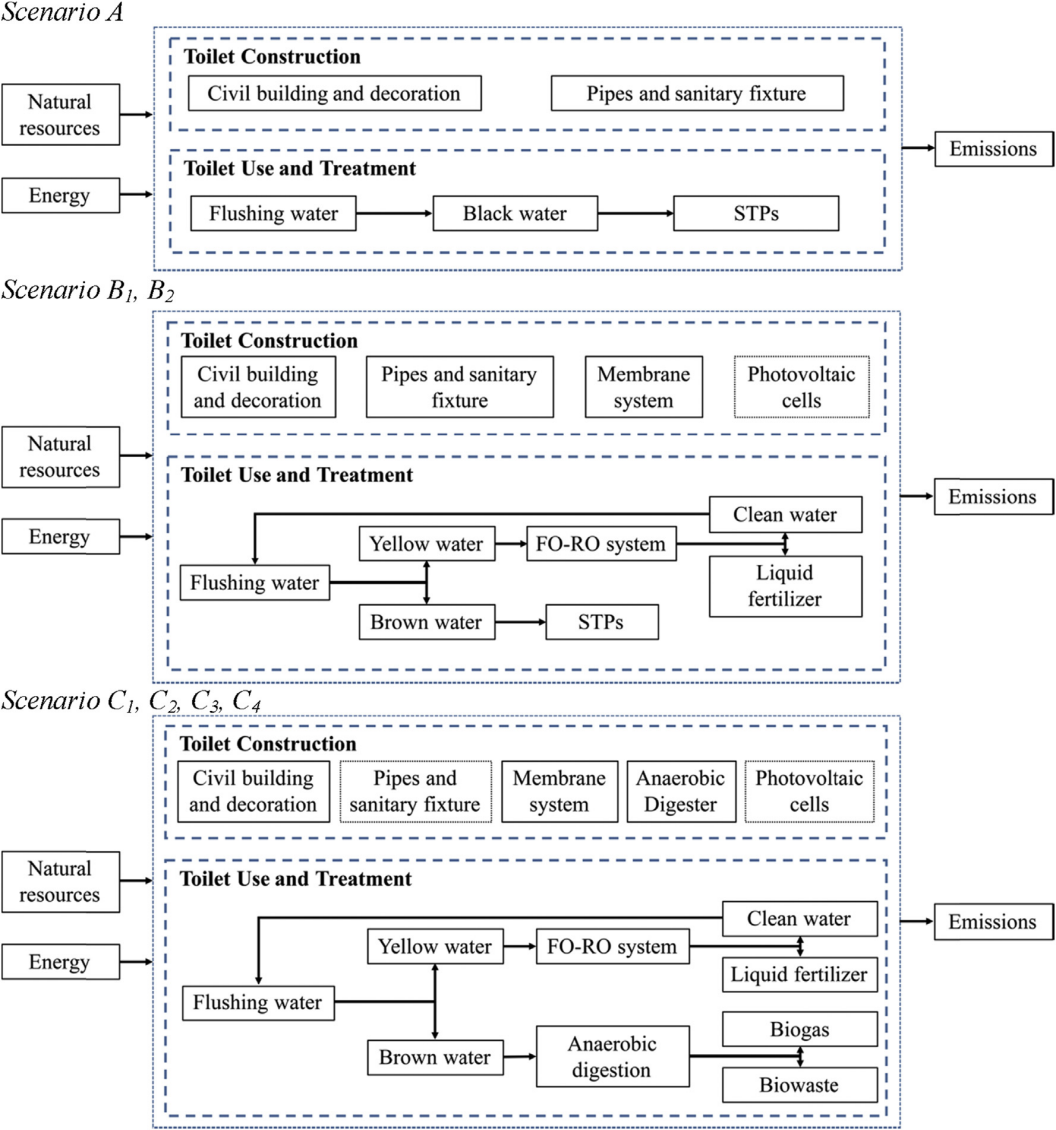
Toilet system boundaries in the study.

All designed toilet systems were equipped with 6 closet pans for the female and 2 closet pans and 2 urinals for the male which would be built in a park or scenic spot, serving 780 women and 800 men every day based on the standards for design of public toilets, *i.e. Chinese Standard for design of urban public toilets (CJJ 14–2016)*; *Beijing Specification for construction of public toilets (DB1*1/T *190–2016)*. As all scenarios are designed for daily use, the functional unit was defined as “collecting and treating the human waste of 780 women and 800 men in one-day toilet use”. The system boundaries are shown in Fig. 1. From the pilot-scale application project mentioned in our preliminary research, it can be inferred that scenario A can produce 3344.325 L mixed urine and feces (black water) per day while scenario B_1_-C_4_ can produce 395.001 L urine (yellow water) and 368.662 L feces (brown water) per day. More detailed design data is shown in Table A1. (supplementary materials).

### 3.2 Life cycle cost-benefit analysis

Any economic activity of human society, including policies and projects, will have an impact on the allocation of the environment and natural resources. It is necessary to assess these impacts to determine whether a policy should be enacted, or whether a project should be developed and constructed. For most government agencies and international agencies, the cost-benefit analysis has been used as the main evaluation method to assess the environmental impact through the whole life of the project, and then social concerns about the environment are included into the feasibility study of the project (Belli, 2001). The impact of transfer payments and internalize external effects such as indirect costs and benefits needs to be excluded during analysis process in order to comprehensively assess the economics of the project from the perspective of the entire country and society. The economic net present value (ENPV) is an indicator to compare different project from economic perspective. It is the sum of the present value of the construction starting point converted from the net benefit of each year in the calculation period of the project based on the social discount rate. The project with a higher ENPV is better and more feasible when comparing different projects (Boardman, 2001). It can be calculated by the following equation (1):

(1) $$\text{ENPV}=\sum_{t=0}^{20}\left[\left(B_{t}-C_{t}\right)/{\left(1+i\right)}^{t}\right]$$

where *t* = years; *B*_*t*_ = benefit for *t*; *C*_*t*_ = cost for *t*; *i* = social discount rate. The analysis period is 20 years in this study, which is typical for toilets works in China. According to the requirement of *Ministry of Housing and Urban-Rural Development of the People's Republic of China*, social discount rate of long-term environmental protection project should be 8%, thus *i* equals 8% in this study.

The main cost and benefit of these systems during their lifespan were analyzed in Table 3. More detailed calculations can be found in Tables. A3–A6. (supplementary materials). In addition, since some of the external benefits have little or no influence on the results of the calculation, the cost-benefit flows in Table 3 do not fully calculate all indirect benefit, *e.g.* benefit of reducing environmental degradation costs caused by water pollution, improvement of global atmospheric environment resulting from water conservation, improvement of local eco-environmental quality by reducing pollutant emissions *etc.*

**Table 3:** Life cycle cost and benefit of seven scenarios.

Items	SA	SB_1_	SB_2_	SC_1_	SC_2_	SC_3_	SC_4_
**Cost (×10**^**4**^**CNY)**^a^							
Civil building and decoration cost b	20.79	28.35	28.35	31.05	31.05	33.75	33.75
Pipes cost c	0.64	0.59	0.59	0.39	0.39	0.18	0.18
Equipment cost c	0.92	34.47	33.47	38.47	36.47	38.47	36.47
Annual tap water and sewage treatment cost d	0.66	0.046	0.046	0.046	0.046	0.00	0.00
Annual electricity cost d	0.00	0.00	0.44	0.00	0.45	0.00	0.82
Annual operational cost e	1.20	3.14	3.09	3.14	3.04	3.74	3.64
Annual material cost f	0.00	0.40	0.40	0.50	0.50	0.60	0.60
Annual depreciation cost	0.00	1.54	1.49	1.74	1.64	1.74	1.64
Sewage collection and treatment facilities cost g	0.99	−0.88	−0.88	−0.99	−0.99	−0.99	−0.99
**Benefit (×10**^**4**^**CNY)**							
Annual liquid fertilizer benefit h	0.00	11.53	11.53	11.53	11.53	11.53	11.53
Annual biogas benefit h	0.00	0.00	0.00	0.003	0.003	0.003	0.003
Annual biowaste benefit h	0.00	0.00	0.00	0.00	0.00	0.00	0.00

a Currency exchange rate in *Bank of China* at January 28, 2018: 1 CNY = 0.1582 USD.

b 2700 CNY/m^2^, ranges from 2400 to 3000 CNY.

c Based on pilot-scale toilet.

d Price of tap water is 6.0 CNY/m^3^, and price of electricity in Beijing is 0.821 CNY/kWh.

e Includes a 1000 CNY per month for cleaner, a 2000 CNY per year for training (SB_1_-SC_4_), and 5% of the equipment cost for maintenance (SB_1_-SC_4_).

f Material refers to fertilizer additives, disinfectants, yellow water stabilization agents and membrane pollution control agents.

g Comes from statistical results (Table. A.5).

h Price of liquid fertilizer is 2000 CNY/m^3^ (converted according to the content of nitrogen, phosphorus, and potassium). The price of biogas is 0.5–1.0 CNY/m^3^, and the biowaste produced by anaerobic digestion do not have the feasibility of selling because of low content of nitrogen, phosphorus, and potassium.

### 3.3 Life cycle environmental assessment

Life Cycle Assessment (LCA) is a comprehensive method developed to evaluate the potential environmental impacts of a product system throughout its life cycle (ISO 14040, 2006; Klöpffer, 2014). It has been applied to assess sanitation systems to characterize their environmental impacts and evaluate their potential trade-offs, *i.e.* source-separated systems (Remy and Jekel, 2012), rural toilet systems (Gao et al., 2017) and struvite precipitation (Ishii and Boyer, 2015). In this study, LCA was used to compare resource-oriented toilet with conventional toilet from an environment perspective to identify the obstacles and limitations of the resource-oriented toilet in order to conduct more specific research to make it more environmentally sustainable in next stage.

The system boundaries are shown in Fig. 1. The inventory for each scenario was established in spreadsheet format and described in the LCA software Gabi 8.0. All data were collected from experimental performance, reasonable assumption and computer models. More detailed inventory data is shown in Table A2. (supplementary materials).

The life cycle impact assessment was characterized by CML 2001–Apr, in which the comprehensive environmental impacts of all scenarios were described in eleven categories: Global Warming Potential (GWP), Acidification Potential (AP), Eutrophication Potential (EP), Ozone Layer Depletion Potential (ODP), Abiotic Depletion Elements (ADP elements), Abiotic Depletion Fossil (ADP fossil), Freshwater Aquatic Ecotoxicity (FAETP), Human Toxicity Potential (HTP), Marine Aquatic Ecotoxity (MAETP), Photochem. Ozone Creation Potential (POCP), and Terrestric Ecotoxicity Potential (TETP).

## 4 Results and discussion

### 4.1 Economic benefits

Based on Table 3, the ENPV results of each scenario was calculated with equation (1) to compare the economic benefits of seven scenarios, and the results are shown in Table 4.

**Table 4:** The ENPV results of seven scenarios.

Systems	ENPV (×10^4^ CNY)
Scenario A	−41.60
Scenario B_1_	0.35
Scenario B_2_	−1.99
Scenario C_1_	−8.96
Scenario C_2_	−9.42
Scenario C_3_	−17.87
Scenario C_4_	−21.96

As shown in Table 3, the resource-oriented toilet systems (SB_1_-SC_4_) have higher cost in construction and equipment but lower cost in the operation phase when compared to conventional toilet system (SA). Therefore, the ENPV results, shown in Table 4, indicate that SA is not feasible from economic perspective because of the high expenditure for the usage of tap water to flush toilets and the sewage treatment in STPs. However, SB_1_-SC_4_ are more feasible due to the application of FO technology and RO technology to concentrate yellow water, obtaining liquid fertilizer and clean water for flushing.

Due to the requirement of more resource recovery facilities and equipment in SC_1_-SC_4_, SB_1_ and SB_2_ which simply recover nutrients from the urine are more feasible in economic perspective. It is also because the benefits of the recovered heat and other resources were not included when using anaerobic digestion to treat brown water in SC_1_, SC_2_, SC_3_, and SC_4_.

Comparing SB_1_ with SB_2_, SC_1_ with SC_2_, or SC_3_ with SC_4_, it can be demonstrated that the use of photovoltaic cells benefits a little, but there are still uncertainties, because it depends on the production processes and the maintenance consumption during 20 years lifespan. Additionally, the cost of wiring in SB_2_, SC_2_ and SC_4_ is not included in Table 3 because the distance is not easy to estimate and SB_1_, SC_1_ and SC_3_ also need wires in case of rainy season in some area.

Because both SC_3_ and SC_4_ use RO system to generate enough flush water from surface water (*e.g.* river water, lake water, *etc*.), an additional 10 m^3^ tank is required in these two scenarios. Therefore, SC_3_ and SC4 have higher construction costs than SC_1_ and SC_2_, resulting in lower ENPV values as Table 4 shows. However, the ENPV results of SC_3_ and SC_4_ are still much higher than SA. Moreover, SC_3_ can operate completely independently without the need of grid, water supply and drainage systems. Although its ENPV value is negative, it is still a better choice for rural area to collect and treat human waste to decrease investment and recover nutrients as fertilizer.

With the further upgrading and renovation of related technologies, as well as the scale and standardization of supporting equipment and products, the cost of the resource-oriented toilet systems will decrease gradually. Thus, some scenarios (SB_1_, SC_1_ and SC_2_) are reasonable to have positive benefits when full-scale implemented. Furthermore, SB_1_ and SB_2_ are preferred in areas where water supply and sewage treatment infrastructure are available, while SC_1_ and SC_2_ have priority in areas where sewage treatment needs a high cost and SC_3_ can also be applied in areas without any external facilities.

### 4.2 Environmental impacts

The inventory data are shown in Table A2. For each scenario, the energy consumption, emissions and environmental benefits were calculated, and the inventory data in all scenarios were estimated with the experimental data conducted in the pilot-scale resource-oriented toilet. The presented results also include fertilizer offsets based on the mass of nitrogen, phosphorus, and potassium, as one kg of N, P, K from the liquid fertilizer production would offset the equivalent kg of N, P, K in the commercial fertilizer.

**Fig. 2:**
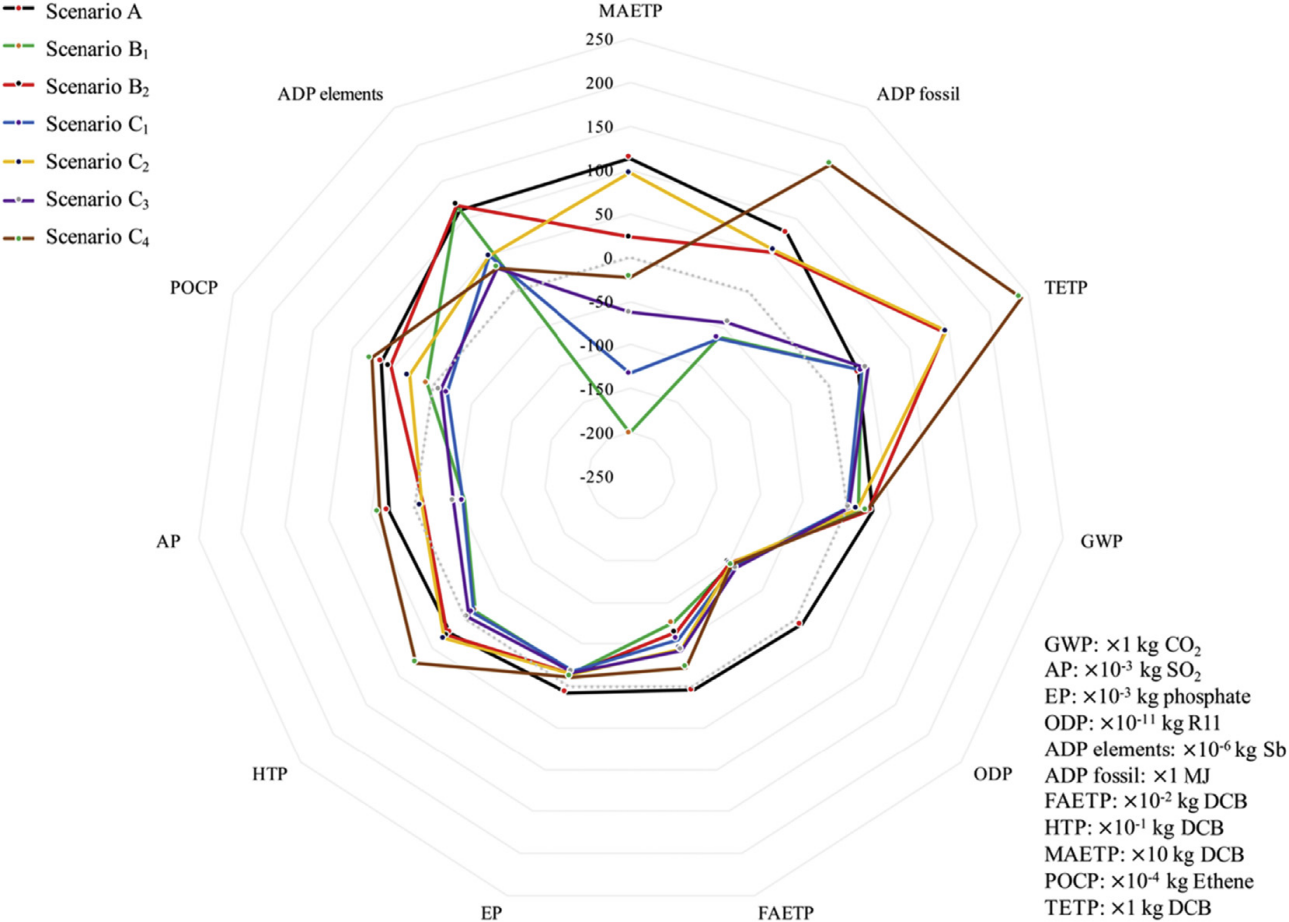
Environmental profiles of seven scenarios.

**Fig. 3:**
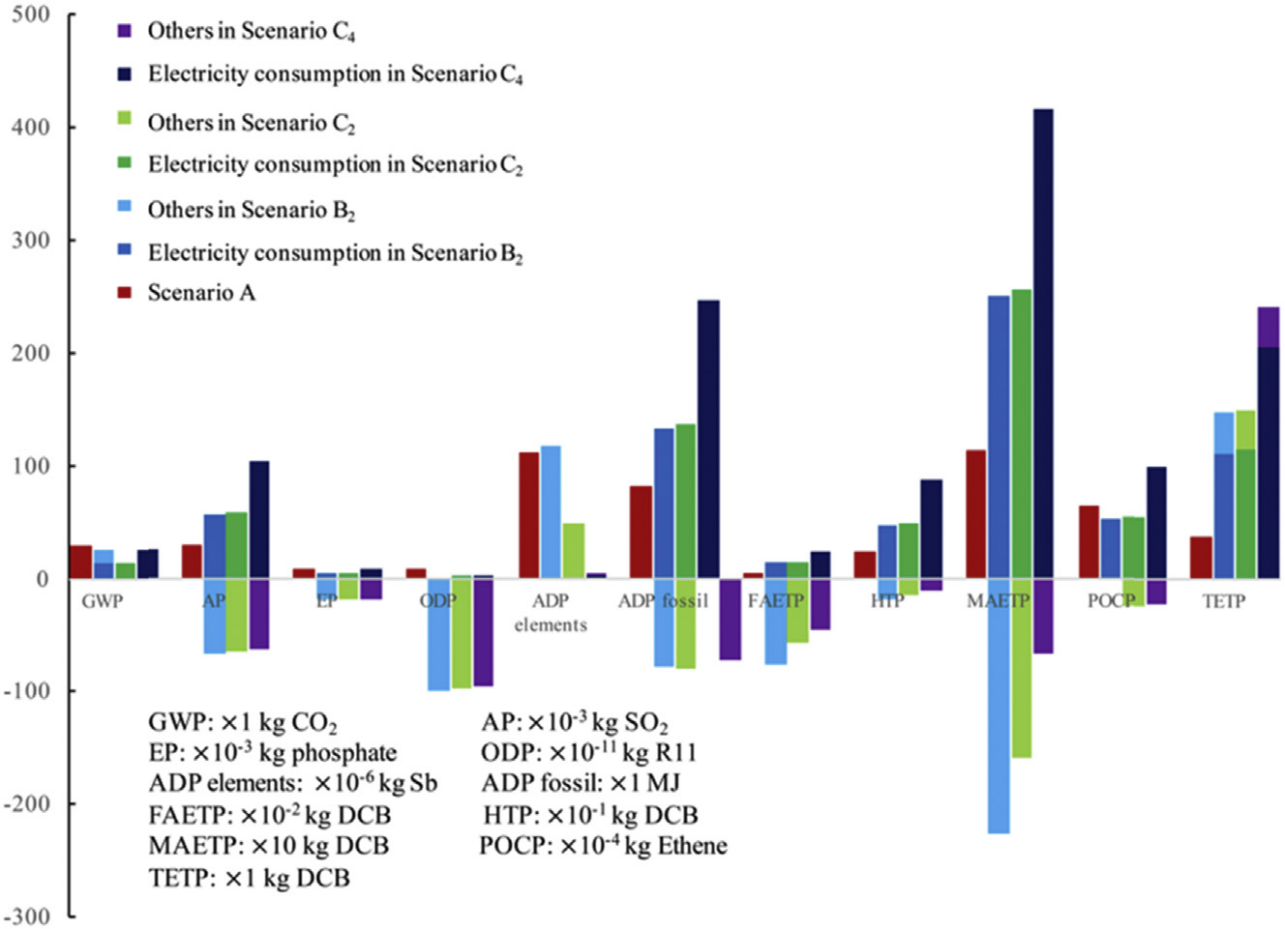
Environmental impacts of electricity consumption in SB_2_, SC_2_ and SC_4_.

Fig. 2 shows the environmental profiles of different scenarios. The environmental impacts of the resource-oriented toilet systems are lower than the conventional toilet system in almost all aspects as indicated in Fig. 3. In some aspects, there are even positive impacts to the environment, *e.g.* MAETP of SB_1_, SC_1_, and SC_3_. However, due to electricity consumption in SB_2_, SC_2_, and SC_4_, the burdens on TETP of these three scenarios are much higher than that of SA. Furthermore, SC_4_ has a higher environment cost in TETP, ADP fossil, HTP, AP, and POCP than other scenarios. Besides, some similar trends can be found between SB_1_ and SC_1_. The reason is that the fertilizer offsets are the same in SB_1_ and SC_1_, and additional biogas and biowaste offsets, as well as heat and nutrients benefits, in SC_1_ which have not been fully recovered. Furthermore, this analysis highlights that SB_1_, SC_1_ and SC_3_, especially SC_1_, are feasible from environmental sustainability.

**Fig. 4:**
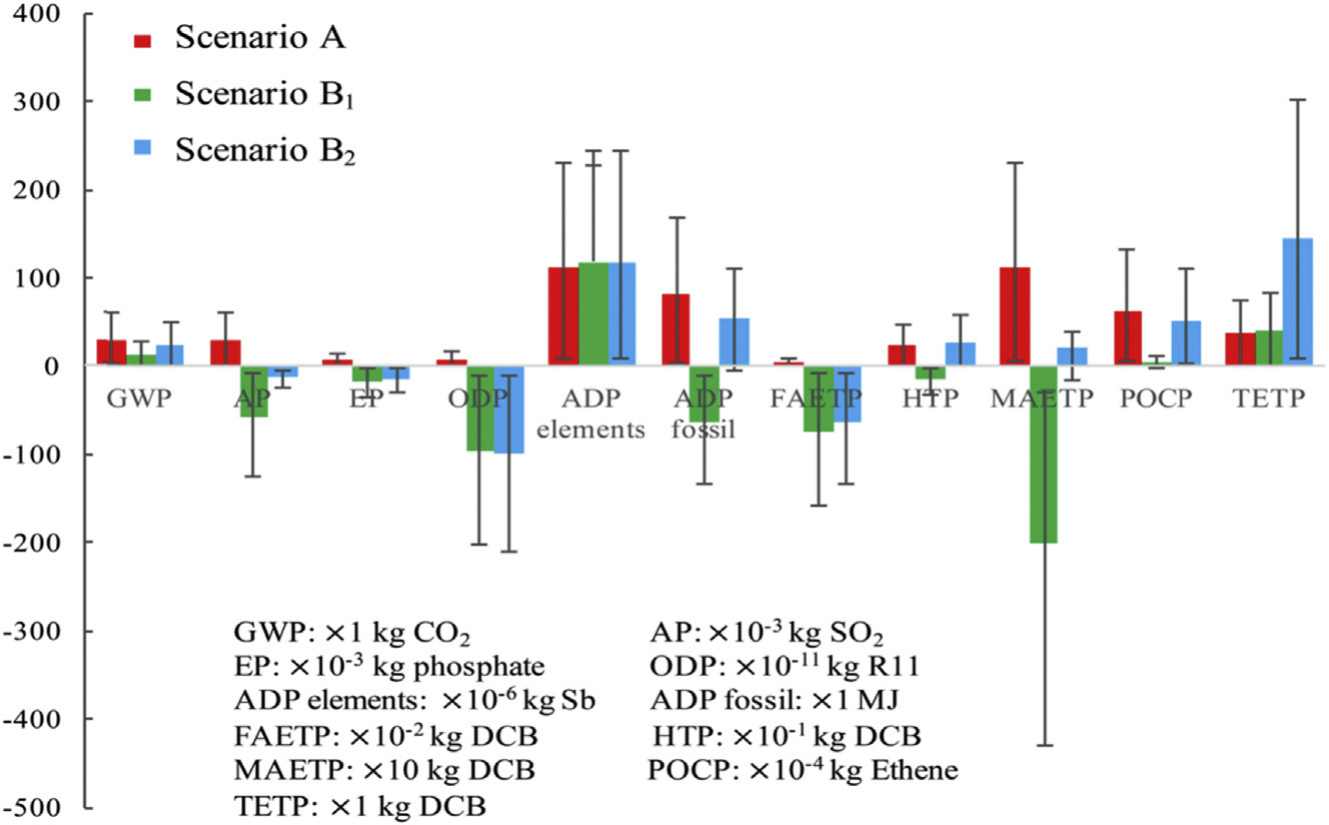
Comparative environmental profiles for SB_1_ and SB_2_.

**Fig. 5:**
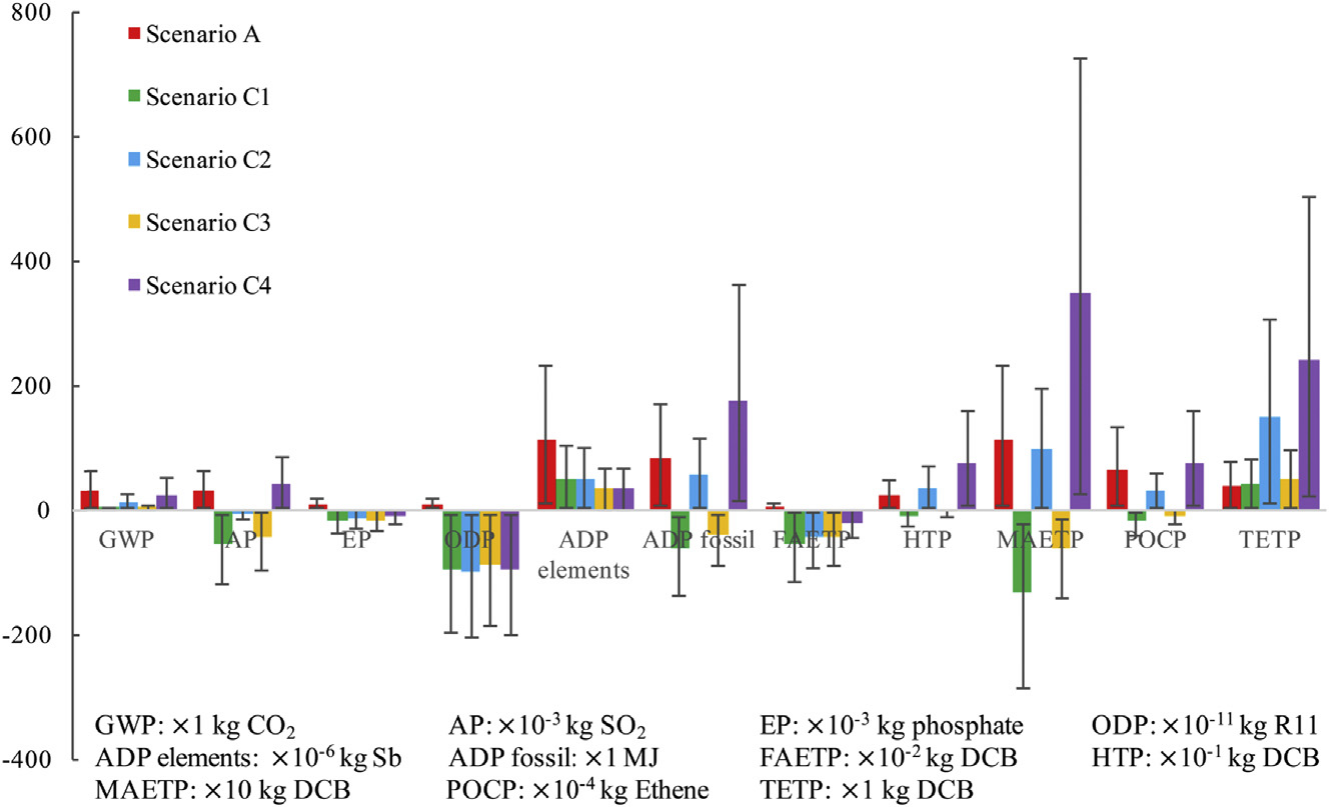
Comparative environmental profiles for SC_1_, SC_2_, SC_3_ and SC_4_.

Further analysis was provided in Fig. 3, Fig. 4, Fig. 5. Fig. 3 provides overall impacts for electricity consumption in the resource-oriented toilet systems. In detail, electricity consumption is the main contributor to GWP (53.6% in SB_2_, 111.4% in SC_2_, 103.1%in SC_4_), AP (−551.5% in SB_2_, 737.5% in SC_2_, 252.0%in SC_4_), EP (−29.0% in SB_2_, 28.2% in SC_2_, -68.2%in SC_4_), ADP fossil (244.8% in SB_2_, 242.4% in SC_2_, 141.7%in SC_4_), FAETP (−20.2% in SB_2_, 29.6% in SC_2_, -107.1%in SC_4_), HTP (166.0% in SB_2_, 146.4% in SC_2_, 114.9%in SC_4_), MAETP (1099.2% in SB_2_, 266.3% in SC_2_, 119.2%in SC_4_), POCP (100.3% in SB_2_, 188.1% in SC_2_, 128.9%in SC_4_) and TETP (76.2% in SB_2_, 76.9% in SC_2_, 84.0%in SC_4_). The results of the environmental impacts include the offsets of the biogas, liquid fertilizer, *etc*. The offsets reduce the total impacts a lot and even turn them to positive impacts to the environment. As results, the contributors of the electricity consumption would be more than 100%, and even result in negative values. Furthermore, the impacts of the electricity consumption in SB_2_, SC_2_, and SC_4_ are even higher than total impacts of SA in some aspect, *e.g.* AP, ADP fossil, FAETP, HTP, MAETP and TETP. In particular, RO system used to concentrate draw solution and produce clean water consumes the largest percent of electricity in the resource-oriented toilet systems (92.3% in SB_1_ and SB_2_, 90.1% in SC_1_ and SC_2_, 94.5% in SC_3_ and SC_4_), and contribute a lot to the environmental impacts. The same result was discussed in other study (Rahman et al., 2016). However, fertilizer offsets in the resource-oriented toilet systems have more positive environmental impacts as shown in Fig. 3, Fig. 4, Fig. 5. It suggests that the resource-oriented toilets with forward osmosis concentrating urine provide a potential solution to sustainable development and decentralized sanitation success (Trimmer et al., 2017).

The benefit of using photovoltaic cells to replace power grid is uncertain because the impacts would be affected by the multi-Si production process and the maintenance consumption during 20 years lifespan (Fu et al., 2015), thus, an overall contrast between SB_1_ with SB_2_ was conducted in Fig. 4, also a contrast between SC_1_, SC_2_ and SC_2_ in Fig. 5. As these two figures shows, whether use photovoltaic cells or not have obvious impacts on GWP, AP, ADP fossil, HTP, MAETP, POCP, TETP. Both Fig. 3, Fig. 4 show the burdens mainly come from the coal-fired power plants. Fig. 4, Fig. 5 suggest that the less treatment processes and emissions in the resource-oriented toilet would result in less environmental harmful as SC_1_ is better than SB_1_ and SC_3_ in almost all aspects. In addition, the transportation of surface water was not included in the inventory of SC_3_ and SC_4_, because it is not reasonable to transport water with trucks in the area without any external facilities, in contrast, it needs workers to replace trucks.

Furthermore, as the inventory data mainly comes from reasonable estimation based on the existing pilot-scale resource-oriented toilet used in Tsinghua Primary School, a lot of external factor will affect the overall uncertainty. For example, the specialized equipment consumes more material and energy in its production process; the composition of urine and feces depends on personal habits a lot, and the children may prefer sweet food like cake, ice cream while the dietary habits and digestion ability of adults may wider and stronger than children. A ±10% deviation was included to the inventory data, which would be affected by the toilet scale, composition of urine and feces, consumption of maintenance during 20 years lifespan and instability of the system, then, the analysis process was conducted and the results were presented as the error bars in Fig. 4, Fig. 5 to show the potential uncertainty ranges.

**Fig. 6:**
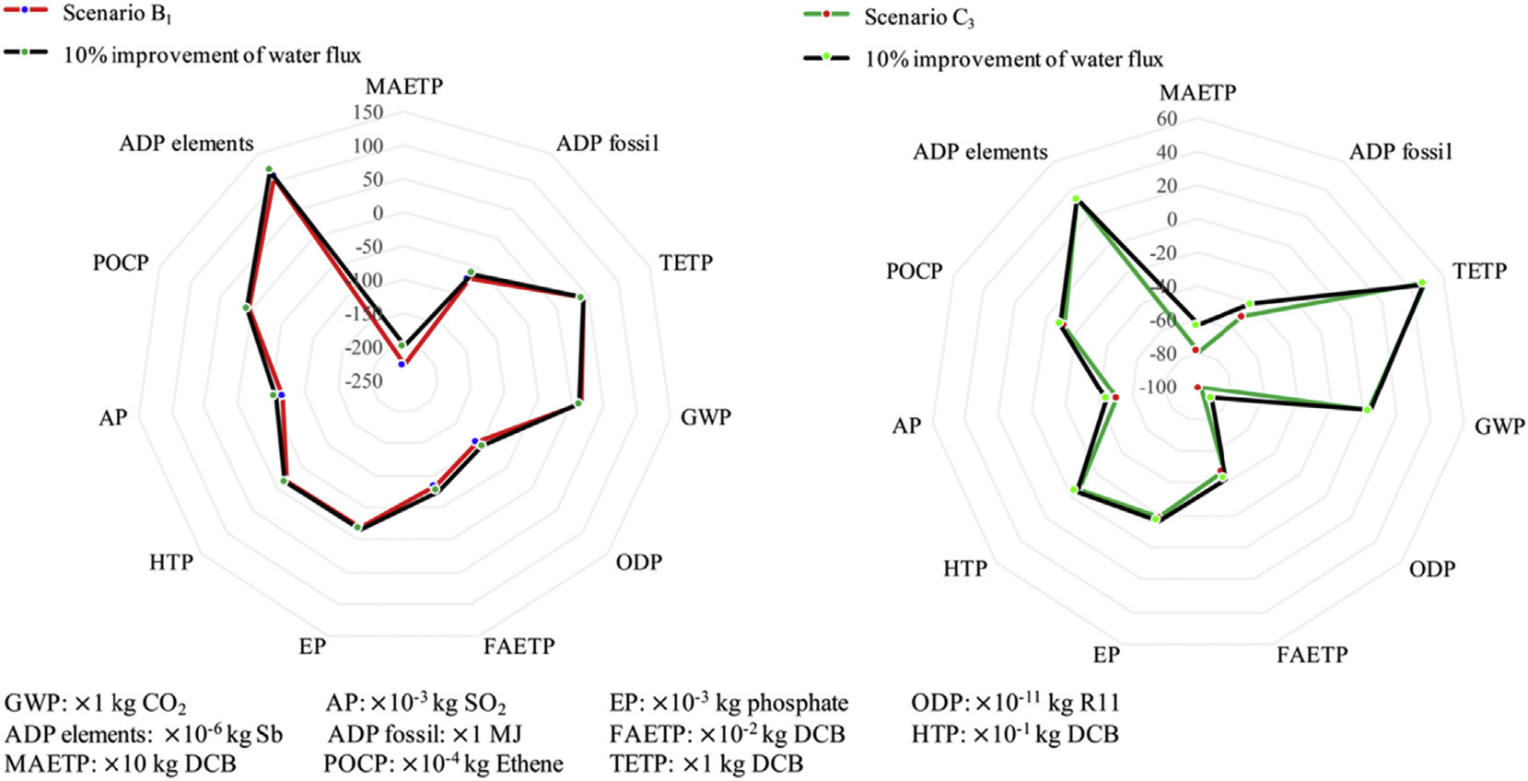
Impacts of 10% improvement of water flux in forward osmosis process on SB_1_ and SC_3_.

A 10% improvement of water flux in forward osmosis process was assumed to evaluate the potential impacts of further development of membrane technology. the new environmental profiles of SB_1_ and SC_3_ are presented in Fig. 6. The little improvement of water flux has obvious impacts on MAETP (14.0% lower in SB_1_, 24.7% lower in SC_3_) and ODP (10.2% lower in SB_1_, 10.1% lower in SC_3_). The high-performance forward osmosis membranes with high water flux and solute rejection rate will benefit a lot for environmental sustainability of resource-oriented toilets.

Another source of uncertainty that was not addressed is the septic tank in scenario A. In China, almost all of the toilets are attached with a septic tank to treat urine and feces as a pretreatment process. The environmental impacts of this process are not easy to evaluate. Thus, this process was ignored and an assumption was made that the urine and feces were transported to STPs straightly through the sewer. As result, the environmental cost would decrease, because much more methane would be produced under anaerobic conditions in the septic tank (Diaz-Valbuena et al., 2011). In contrast, there is no need to build septic tank in other scenarios. It turns toilets from pollution center to resource center.

### 4.3 Trade-offs

As the study aimed to provide support for decision makers to choose which toilet systems to meet different requirements of sustainability, it is necessary to make a multi-criteria decision analysis based on the ENPV results and environmental impacts. Analytic hierarchy process is an effective method to construct a utility function to evaluate the trade-offs between multi-criteria, and has been used for environmental management (Ishizaka and Nemery, 2013; Patra et al., 2018). In this study, a simplified analytic hierarchy process was conducted. The environmental impacts were quantized with equal priorities for each environmental impact categories. The quantification equation was:

(2) $$E_{impact}=\sum_{i=1}^{11}\left[\frac{1}{11}\times \frac{E_{i}}{{\left|E_{i}\right|}_{min}}\right]$$

where *E*_*impact*_ = comprehensive environmental impact of a certain scenario; *i* = different environmental impact categories; *E*_*i*_ = one environmental impact category of a certain scenario; *|E*_*i*_*|*_*min*_ = the absolute minimum of one environmental impact category among all scenarios.

**Fig. 7:**
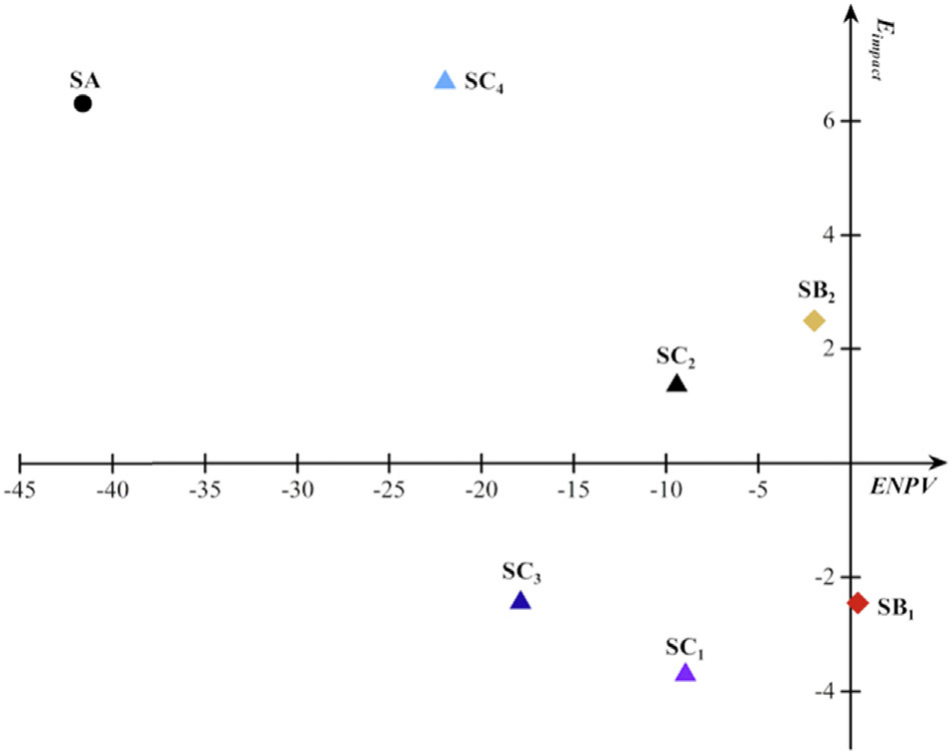
Trade-offs between economic benefits and environmental impacts.

All scenarios were distributed in a X-Y coordinate system according to their ENPV results and environmental impacts in Fig. 7.

As shown in Fig. 7, the environmental cost of SB_2_, SC_2_, SC_4_ are higher than SB_1_, SC_1_, SC_3_. It mainly attributes to the electricity consumption. SB_1_ has the highest ENPV, while SC_1_ have the lowest environmental impacts. The expenditure of SC_3_ and SC_4_ are higher than SC_1_ and SC_2_ because of the usage of RO system to treat surface water to produce flush water. However, as quality standards of flush water is significantly different with RO effluent, the economic and environmental cost will both decrease when other simpler processes were applied to purify surface water such as coagulation, precipitation, chlorine disinfection, etc. Furthermore, the comparison between SB_1_ and SC_1_, or SB_2_ and SC_2_ indicates that the complete resource recovery scenarios with more treatment processes results in a lower environmental impact and a higher economic cost.

Overall, scenario B_1_ is the best choice to replace conventional toilet system with a positive environmental sustainability and economic feasibility. The clean energy such as solar energy, wind energy, *etc.* could support the full-application of these resource-oriented toilet systems in further expansion.

## 5 Conclusions

In this study, the resource-oriented toilet systems proved to have more benefits in economic and environmental aspects after compared with the conventional toilet system. It could be applied in both urban and rural areas to achieve the optimal solution for pollution control, resource recovery and economic effectiveness. The results indicate that resource-oriented toilets (2 urinals and 8 closet pans for 20 years use) with forward osmosis technology concentrating urine have 20–43 × 10^4^ CNY improvement in ENPV results. The liquid fertilizer production in resource-oriented toilet systems offset a lot of environmental impacts as it provides an alternative choice for agriculture besides commercial fertilizer production. However, the energy consumption in resource-oriented toilets contributes a lot to the environmental impacts. Taking both economic feasibility and environmental sustainability into consideration, the partial resource-oriented toilet (SB_1_) is the best choice while the totally independent resource-oriented toilet (SC_3_) could be applied to replace conventional toilets in rural area without any external facilities (*i.e.* water supply and drainage system, sewage treatment system, grid, *etc.*).

The uncertainty mainly come from the application of specialized equipment, fluctuation of urine and feces composition, and instability of systems. More researches are needed to improve the efficiency of the membrane system. The improvement of forward osmosis performance and the decrease of energy consumption can both benefit a lot. Moreover, more effort is needed to investigate the technical feasibility of the resource-oriented toilet systems in rural area. If the new toilet systems could be more accepted by users in different areas, the manufacturing cost will be lower. Simultaneously, more users use the toilet, more liquid fertilizer offsets produce.
