# Supplementary material for: Life cycle cost and environmental assessment for resource-oriented toilet systems
Source: J Clean Prod. 2018 Sep 20;196:1188–97. doi: 10.1016/j.jclepro.2018.06.129 (PMC6106690; doi:10.1016/j.jclepro.2018.06.129)
Supplement: Multimedia component 1 [file mmc1.docx]

Appendices

**Table List:**

**Table A.1.** Detailed data of different toilet systems

**Table A.2.** Inventory for different toilet systems

**Table A.3.** Cost and Benefit of seven scenarios

**Table A.4.** Pipe cost

**Table A.5.** Wastewater treatment cost

**Table A.6.** The ENPV calculation process. (×10^4^ CNY)

**Figure list:**

**Fig. A.1.** Simplified schematic of Scenario A

**Fig. A.2.** Simplified schematic of Scenario B

**Fig. A.3.** Simplified schematic of Scenario C

**Table A.1.** Detailed data of different toilet systems

| **Items** | **Scenario A** | **Scenario B_1_-C_4_** |
| --- | --- | --- |
| Urinals for male | 2 | 2 |
| Closet pans for male | 2 | 2 |
| Closet pans for female | 6 | 6 |
| Toilet area (m^2^) | 75 | 75 |
| Tools area (m^2^) | 2 | 30-50 *^a^* |
| Male service (person/d) | 800 | 800 |
| Female service (person/d) | 780 | 780 |
| Toilet flush volume (L) | 4 | 1.2 |
| Urinal flush volume (L) | 1.5 | 0.1 |
| Stool frequency (times/d) | 263.33 | 263.33 |
| Urine frequency (times/d) | 1316.7 | 1316.7 |
| Black water production (L/d) | 3344.325 | - |
| Yellow water production (L/d) | - | 395.001 |
| Brown water production (L/d) | - | 368.662 |

*^a^* SB_1_ and SB_2_ are 30 m^2^; SC_1_ and SC_2_ are 40 m^2^; SC_3_ and SC_4_ are 50 m^2^

All design data is based on the standards for toilet construction, *i.e.* Standard for design of urban public toilets CJJ 14-2016; Specification for construction of public toilets DB11/T 190-2016. A ratio of 1:5 was chosen to determine the ratio of stool to urine in this study, which is typical in our daily life. Besides, the volume of feces and urine each time are both 0.2L respectively. The calculation process is as follows.

Stool frequency:

$$\left( 780+800 \right)\times\frac{1}{6}=263.33$$

Urine frequency:

$$\left( 780+800 \right)\times\frac{5}{6}=1316.67$$

Black water production (Scenario A):

$$263.33\times\left( 4+0.2 \right)+1316.67\times\left( 1.5+0.2 \right)=3344.325 L/d$$

Yellow water production (Scenario B_1_-C_4_):

$$1316.67\times\left( 0.1+0.2 \right)=395.001 L/d$$

Brown water production (Scenario B_1_-C_4_):

$$263.33\times\left( 1.2+0.2 \right)=368.662 L/d$$

**Table A.2.** Inventory for different toilet systems

| **Items** | **Unit** | **Scenario A** | **Scenario B_1_** | **Scenario B_2_** | **Scenario C_1_** | **Scenario C_2_** | **Scenario C_3_** | **Scenario C_4_** |
| --- | --- | --- | --- | --- | --- | --- | --- | --- |
| Construction Area | m^2^ | 77 | 105 | 105 | 115 | 115 | 125 | 125 |
| Toilet construction for 1 day use | | | | | | | |  |
| Water | kg | 0.10136588 | 0.13822620 | 0.13822620 | 0.15139060 | 0.15139060 | 0.16455500 | 0.16455500 |
| Cement | kg | 0.80227686 | 1.09401390 | 1.09401390 | 1.19820570 | 1.19820570 | 1.30239750 | 1.30239750 |
| Steel | kg | 0.85396157 | 1.16449305 | 1.16449305 | 1.27539715 | 1.27539715 | 1.38630125 | 1.38630125 |
| Cast iron | kg | 0.09503725 | 0.12959625 | 0.12959625 | 0.14193875 | 0.14193875 | 0.15428125 | 0.15428125 |
| Gravel | kg | 0.11782078 | 0.16066470 | 0.16066470 | 0.17596610 | 0.17596610 | 0.19126750 | 0.19126750 |
| Lime | kg | 0.09503725 | 0.12959625 | 0.12959625 | 0.14193875 | 0.14193875 | 0.15428125 | 0.15428125 |
| Wooden frame | kg | 0.02109569 | 0.02876685 | 0.02876685 | 0.03150655 | 0.03150655 | 0.03424625 | 0.03424625 |
| Float glass | kg | 0.11064823 | 0.15088395 | 0.15088395 | 0.16525385 | 0.16525385 | 0.17962375 | 0.17962375 |
| Facing brick | kg | 0.38647686 | 0.52701390 | 0.52701390 | 0.57720570 | 0.57720570 | 0.62739750 | 0.62739750 |
| Steel pipe | kg | 0.12400900 | 0.12400900 | 0.12400900 | 0.02682740 | 0.02682740 | - | - |
| PP pipe | kg | 0.00060986 | 0.00081315 | 0.00081315 | 0.00081315 | 0.00081315 | 0.00081315 | 0.00081315 |
| PB pipe | kg | 0.00658356 | - | - | - | - | - | - |
| PVC pipe |  | - | 0.01075070 | 0.01075070 | 0.01075070 | 0.01075070 | 0.01075070 | 0.01075070 |
| Quartz sand | kg | 0.00024658 | 0.00024658 | 0.00024658 | 0.00024658 | 0.00024658 | 0.00024658 | 0.00024658 |
| Clay | kg | 0.01331510 | 0.01331510 | 0.01331510 | 0.01331510 | 0.01331510 | 0.01331510 | 0.01331510 |
| Kaolin | kg | 0.00715068 | 0.00715068 | 0.00715068 | 0.00715068 | 0.00715068 | 0.00715068 | 0.00715068 |
| Limestone | kg | 0.00024658 | 0.00024658 | 0.00024658 | 0.00024658 | 0.00024658 | 0.00024658 | 0.00024658 |
| Feldspar | kg | 0.00591781 | 0.00591781 | 0.00591781 | 0.00591781 | 0.00591781 | 0.00591781 | 0.00591781 |
| Electricity | MJ | 0.07394300 | 0.07394300 | 0.07394300 | 0.07394300 | 0.07394300 | 0.07394300 | 0.07394300 |
| Diesel | kg | 0.00010208 | 0.00010208 | 0.00010208 | 0.00010208 | 0.00010208 | 0.00010208 | 0.00010208 |
| Natural Gas | kg | 0.00851297 | 0.00851297 | 0.00851297 | 0.00851297 | 0.00851297 | 0.00851297 | 0.00851297 |
| Toilet use and sewage treatment (or nutrients recovery) for 1 day use | | | | | | | |  |
| Water for flush | kg | 3028.325 | 210.662 | 210.662 | 210.662 | 210.662 | - | - |
| Electricity consumed in STPs | kWh | 1.02670931 | 0.1131792 | 0.1131792 | - | - | - | - |
| Ferrous sulphate consumed in STPs | kg | 2.53366 | 0.279298 | 0.279298 | - | - | - | - |
| Sludge for landfill | kg | 26.3757 | 20.4436 | 20.4436 | - | - | - | - |
| CH_4_ emission | kg | 0.67530 | 0.47386761 | 0.47386761 | - | - | - | - |
| N_2_O emission | kg | 0.01939 | 0.00531892 | 0.00531892 | - | - | - | - |
| Biogas | kg | - | - | - | 0.135041 | 0.135041 | 0.135041 | 0.135041 |
| Biowaste for agriculture | kg | - | - | - | 352.8622 | 352.8622 | 352.8622 | 352.8622 |
| Energy for use | kWh | - | 0.79 | 0.79 | 0.79 | 0.79 | 0.79 | 0.79 |
| Energy for FO system | kWh | - | 0.316008 | 0.316008 | 0.316008 | 0.316008 | 0.316008 | 0.316008 |
| Energy for RO system | kWh | - | 13.62069 | 13.62069 | 13.62069 | 13.62069 | 25.72759 | 25.72759 |
| Energy for maintenance | kWh | - | 0.02740 | 0.02740 | 0.02740 | 0.02740 | 0.02740 | 0.02740 |
| Energy for anaerobic digester | kWh | - | - | - | 0.36 | 0.36 | 0.36 | 0.36 |
| Electricity for use and treatment | kWh | - | - | 14.754098 | - | 15.114098 | - | 27.220998 |
| Photovoltaic cell | kWp | - | 0.000336852 | - | 0.000345072 | - | 0.000621484 | - |
| Membrane | kg | - | 0.001084640 | 0.001084640 | 0.001084640 | 0.001084640 | 0.001084640 | 0.001084640 |
| Polyethylene | kg | - | 0.000545479 | 0.000545479 | 0.000545479 | 0.000545479 | 0.000545479 | 0.000545479 |
| Water for backwash | kg | - | 1.2 | 1.2 | 1.2 | 1.2 | 1.2 | 1.2 |
| Sodium hypochlorite | kg | - | 0.000016438 | 0.000016438 | 0.000016438 | 0.000016438 | 0.000016438 | 0.000016438 |
| Citric acid | kg | - | 0.000657534 | 0.000657534 | 0.000657534 | 0.000657534 | 0.000657534 | 0.000657534 |
| Sodium chloride | kg | - | 0.000780000 | 0.000780000 | 0.000780000 | 0.000780000 | 0.000780000 | 0.000780000 |
| Water for draw solution | kg | - | 7.19 | 7.19 | 7.19 | 7.19 | 7.19 | 7.19 |
| Nitrogen fertilizer production | kg | - | 3.3654 | 3.3654 | 3.3654 | 3.3654 | 3.3654 | 3.3654 |
| Phosphorous fertilizer production | kg | - | 0.2528 | 0.2528 | 0.2528 | 0.2528 | 0.2528 | 0.2528 |
| Potassium fertilizer production | kg | - | 1.55434 | 1.55434 | 1.55434 | 1.55434 | 1.55434 | 1.55434 |

All data were collected from experimental performance, reasonable assumption and computer models. Furthermore, divided all the data by 7300 to get the inputs and outputs of one-day use.

Some calculations in Table. A.2 are shown below:

Concentration rate in FO process: 2.5

Water flux of FO process: 2$L/\left( h\cdot m^{2} \right)$

FO membrane area: $0.25\times150=37.5 m^{2}$

Water flux of RO process: 8$L/\left( h\cdot m^{2} \right)$

RO membrane area: 8.7 m^2^

Nutrients recovery:

Liquid fertilizer: $395.001L_{YW}\times\frac{1}{2.5}=158.000 L$

Clean water: $395.001L_{YW}\times(1-\frac{1}{2.5})=237.001 L$

Water for flush:

Scenario A: $1316.67\times1.5 L+263.33\times4 L=3028.325 L/d$

Scenario B_1_-C_2_: $1316.67\times0.1+263.33\times1.2-237.001=210.6624L/d$

CH_4_ emission:

Scenario A: $3344.325 L\times8.077\frac{g_{organic matter}}{L}\times0.25\frac{{kg}_{{CH}_{4}}}{{kg}_{organic matter}}=0.6735kg$

Scenario B_1_, B_2_: $368.662 L\times51.41\frac{g_{organic matter}}{L}\times0.25\frac{{kg}_{{CH}_{4}}}{{kg}_{organic matter}}=0.4739kg$

N_2_O emission:

Scenario A: $3344.325 L\times0.7379\frac{g_{TN}}{L}\times0.005\frac{{kg}_{N_{2}O}}{{kg}_{TN}}\times\frac{44}{28}=0.01939kg$

Scenario B_1_, B_2_: $368.662 L\times1.836\frac{g_{TN}}{L}\times0.005\frac{{kg}_{N_{2}O}}{{kg}_{TN}}\times\frac{44}{28}=0.005319kg$

Energy consumption in FO process:

Scenario B_1_-C_3_: $\frac{395.001L_{YW}}{2L/\left( h\cdot m^{2} \right)\times37.5m^{2}}\times60 w=0.316008 kWh$

Energy consumption in RO process:

Scenario B_1_-C_2_: $\frac{237.001L_{draw solution}}{8L/\left( h\cdot m^{2} \right)\times8.7m^{2}}\times4 kw=13.62075 kWh$

Scenario C_3_: $\frac{447.663L_{draw solution}}{8L/\left( h\cdot m^{2} \right)\times8.7m^{2}}\times4 kw=25.72776 kWh$

Photovoltaic cell:

Scenario B_1_: $\frac{0.79kw+0.316008kw+13.62069kw+0.02740kw}{6h\cdot7300d}=0.000336852 kWp/d$

Scenario C_1_: $\frac{0.79kw+0.316008kw+13.62069kw+0.02740kw+0.36kw}{6h\cdot7300d}=0.000345072 kWp/d$

Scenario C_3_: $\frac{0.79kw+0.316008kw+25.72759kw+0.02740kw+0.36kw}{6h\cdot7300d}=0.00062148 kWp/d$

**Table A.3.** Cost and Benefit of seven scenarios

| **Items** | **SA** | **SB_1_** | **SB_2_** | **SC_1_** | **SC_2_** | **SC_3_** | **SC_4_** |
| --- | --- | --- | --- | --- | --- | --- | --- |
| ***Cost (×10^4^ CNY)*** *^a^* | | | | | | | |
| Civil building and decoration cost | 20.79 | 28.35 | 28.35 | 31.05 | 31.05 | 33.75 | 33.75 |
| Pipes cost | 0.64 | 0.59 | 0.59 | 0.39 | 0.39 | 0.18 | 0.18 |
| Equipment cost | 0.92 | 34.47 | 33.47 | 38.47 | 36.47 | 38.47 | 36.47 |
| Annual tap water and sewage treatment cost | 0.66 | 0.046 | 0.046 | 0.046 | 0.046 | 0.00 | 0.00 |
| Annual Electricity cost | 0.00 | 0.00 | 0.44 | 0.00 | 0.45 | 0.00 | 0.82 |
| Annual operational cost | 1.20 | 3.14 | 3.09 | 3.14 | 3.04 | 3.74 | 3.64 |
| Annual material cost | 0.00 | 0.40 | 0.40 | 0.50 | 0.50 | 0.60 | 0.60 |
| Annual depreciation cost | 0.00 | 1.54 | 1.49 | 1.74 | 1.64 | 1.74 | 1.64 |
| Sewage collection and treatment facilities cost | 0.99 | -0.88 | -0.88 | -0.99 | -0.99 | -0.99 | -0.99 |
| ***Benefit (×10^4^ CNY)*** | | | | | | | |
| Annual liquid fertilizer benefit | 0.00 | 11.53 | 11.53 | 11.53 | 11.53 | 11.53 | 11.53 |
| Annual biogas benefit | 0.00 | 0.00 | 0.00 | 0.003 | 0.003 | 0.003 | 0.003 |
| Annual biowaste benefit | 0.00 | 0.00 | 0.00 | 0.00 | 0.00 | 0.00 | 0.00 |
| *^a^* Currency exchange rate in *Bank of China* at January 28, 2018: 1 CNY= 0.1582 USD. | | | | | | | |

Some calculations in Table. A.3 are shown below:

Civil building and decoration cost:

According to Standard for design of urban public toilets CJJ 14-2016 and Specification for construction of public toilets DB11/T 190-2016, the construction area for each squat set is 7.5 m^2^, and the tool room is 2 m^2^. Besides, there is a 30 m^2^ equipment room in SB_1_ and SB_2_, a 40 m^2^ equipment room in SC_1_- SC_4_, another 10 m^3^ (10 m^2^ $\times$ 1 m) reservoir in SC_3_ and SC_4_. As estimated in actual project, the cost of civil building and decoration is 2,700 CNY/m^2^.

SA: $\left( 7.5\frac{m^{2}}{squat set}\times{10}_{squat sets}+2 m^{2} \right)\times2700 \frac{CNY}{m^{2}}=20.79\times{10}^{4} CNY$

SB_1_, SB_2_:$\left( 7.5\frac{m^{2}}{squat set}\times{10}_{squat sets}+30 m^{2} \right)\times2700 \frac{CNY}{m^{2}}=28.35\times{10}^{4} CNY$

SC_1_, SC_2_:$\left( 7.5\frac{m^{2}}{squat set}\times{10}_{squat sets}+40 m^{2} \right)\times2700 \frac{CNY}{m^{2}}=31.05\times{10}^{4} CNY$

SC_3_, SC_4_:$\left( 7.5\frac{m^{2}}{squat set}\times{10}_{squat sets}+50 m^{2} \right)\times2700 \frac{CNY}{m^{2}}=33.75\times{10}^{4} CNY$

Pipe cost:

All data were based on commercial price.

**Table A.4.** Pipe cost

| Items | Cost (CNY/m) | SA (m) | SB_1_ (m) | SB_2_ (m) | SC_1_ (m) | SC_2_ (m) | SC_3_ (m) | SC_4_ (m) |
| --- | --- | --- | --- | --- | --- | --- | --- | --- |
| DN15 PP pipe for urinal water supply | 20 | 3×2 | 4×2 | 4×2 | 4×2 | 4×2 | 4×2 | 4×2 |
| DN50 PB pipe for urinal sewage discharge | 23 | 3×2 | - | - | - | - | - | - |
| DN50 PVC pipe for urinal sewage discharge | 6.5 | - | 4×2 | 4×2 | 4×2 | 4×2 | 4×2 | 4×2 |
| DN25 PP pipe for stool water supply | 30 | 3×8 | 4×8 | 4×8 | 4×8 | 4×8 | 4×8 | 4×8 |
| DN100 PB pipe for stool sewage discharge | 58 | 3×8 | - | - | - | - | - | - |
| DN100 PVC pipe for stool sewage discharge | 21 | - | 4×8 | 4×8 | 4×8 | 4×8 | 4×8 | 4×8 |
| DN100 steel water supply pipe | 38 | 18 | 18 | 18 | 18 | 18 | - | - |
| DN200 steel sewage discharge pipe | 87 | 22.5 | 22.5 | 22.5 | - | - | - | - |
| Municipal pipeline construction cost (CNY) | - | 1,400 | | | | | - | - |
| Total pipe cost  (×10^4^ CNY) | - | 0.64 | 0.59 | 0.59 | 0.39 | 0.39 | 0.18 | 0.18 |

Equipment cost:

Equipment cost = toilet cost + vacuum collecting chamber cost + anaerobic digester cost + membrane system cost + photovoltaic cells cost

The cost for conventional toilet is 1,000 CNY/closet pan, and 600 CNY/urinal. The cost for vacuum urine diversion toilet is 4,500 CNY/closet pan, and 600 CNY/urinal. The total cost for vacuum collecting chamber and membrane system is 297,500 CNY. The cost for 1.5 m^3^ anaerobic digester (AD) is 15,000 CNY. The cost for a group of photovoltaic cell (PV) is 10,000 CNY. All data were estimated by our pilot-scale toilet and based on commercial price.

SA: $1000\frac{CNY}{closet pan}\times8+600\frac{CNY}{urinal}\times2=9200 CNY$

SB_1_: $4500\frac{CNY}{closet pan}\times8+600\frac{CNY}{urinal}\times2+297500CNY+10000CNY=34.47\times{10}^{4} CNY$

SB_2_: $4500\frac{CNY}{closet pan}\times8+600\frac{CNY}{urinal}\times2+297500CNY=33.47\times{10}^{4} CNY$

SC_1_, SC_3_: $4500\frac{CNY}{closet pan}\times8+600\frac{CNY}{urinal}\times2+297500CNY+15000\frac{CNY}{AD}\times2+10000\frac{CNY}{PV}\times2=38.47\times{10}^{4} CNY$

SC_2_, SC_4_: $4500\frac{CNY}{closet pan}\times8+600\frac{CNY}{urinal}\times2+297500CNY+15000\frac{CNY}{AD}\times2+=36.47\times{10}^{4} CNY$

Annual tap water and sewage treatment cost:

The price of tap water is 6.0 CNY/m^3^, including sewage treatment fees.

SA: $3.03\frac{m^{3}}{d}\times365d\times6.0\frac{CNY}{m^{3}}=6635.7 CNY$

SB_1_, SB_2_, SC_1_, SC_2_: $0.21\frac{m^{3}}{d}\times365d\times6.0\frac{CNY}{m^{3}}=459.9 CNY$

Annual electricity cost:

The price of electricity in Beijing is 0.821 CNY/kWh. The annual electricity cost includes only the electricity consumed in treatment process, excluding the energy for lighting, cleaning and so on.

SB_2_: $14.75\frac{kWh}{d}\times0.821\frac{CNY}{kWh}\times365d=4420 CNY$

SC_2_: $15.11\frac{kWh}{d}\times0.821\frac{CNY}{kWh}\times365d=4528 CNY$

SC_4_: $27.22\frac{kWh}{d}\times0.821\frac{CNY}{kWh}\times365d=8157 CNY$

Annual operational cost:

The annual operational cost includes a 1000 CNY per month for cleaner, a 500 CNY per season for transportation of the dry matter of the brown water (scenario B_1_-B_2_), a 2000 CNY per year for training (scenario B_1_-C_4_), and 5% of the equipment cost (excluding toilet cost) for maintenance (scenario B_1_-C_4_). In scenario C_3_ and C_4_, there is a 500 CNY/month expenditure for surface water collection, transportation and storage.

SA: $1000\frac{CNY}{month}\times12=12000 CNY$

SB_1_: $1000\frac{CNY}{month}\times12+2000+307500\times5\%+500\times4=31375 CNY$

SB_2_: $1000\frac{CNY}{month}\times12+2000+297500\times5\%+500\times4=30875 CNY$

SC_1_: $1000\frac{CNY}{month}\times12+2000+347500\times5\%=31375 CNY$

SC_2_: $1000\frac{CNY}{month}\times12+2000+327500\times5\%=30375 CNY$

SC_3_: $1000\frac{CNY}{month}\times12+2000+347500\times5\%+500\frac{CNY}{month}\times12=37375 CNY$

SC_4_: $1000\frac{CNY}{month}\times12+2000+327500\times5\%+500\frac{CNY}{month}\times12=36375 CNY$

Annual material cost:

Material refers to fertilizer additives, disinfectants, yellow water stabilization agents and membrane pollution control agents. All costs were estimated by our pilot-scale toilet.

Annual depreciation cost:

As the key equipment of the system was made of ceramic and metal material, depreciation cost was calculated with the accounting method of annualized average discount amount, and the period is 20 years.

SB_1_: $307500\times5\%=15375 CNY$

SB_2_: $297500\times5\%=14875 CNY$

SC_1_: $347500\times5\%=17375 CNY$

SC_2_: $327500\times5\%=16375 CNY$

SC_3_: $347500\times5\%=17375 CNY$

SC_4_: $327500\times5\%=16375 CNY$

Sewage collection and treatment facilities cost:

The sewage collection and treatment facilities cost came from statistical results. As Table. A.5 shows, the Average cost for treating 1 m^3^/d wastewater is 2974.15 CNY. Among the scenarios, just scenario A needs this cost while other scenarios turn cost into benefit.

SA: $3.34\frac{m^{3}}{d}\times2974.15 \frac{CNY}{{m^{3}}/d}=9933 CNY$

SB_1_, SB_2_: $-\left( 3.34-0.37 \right)\frac{m^{3}}{d}\times2974.15 \frac{CNY}{{m^{3}}/d}=-8833 CNY$

SC_1_, SC_2_, SC_3_, SC_4_: $-3.34\frac{m^{3}}{d}\times2974.15 \frac{CNY}{{m^{3}}/d}=-9933 CNY$

**Table A.5.** Wastewater treatment cost *^a^*

| **Locations** | **Date** | **Capacity (×10^4^ m^3^/d)** | **Total cost (×10^4^ CNY)** | **Cost for treating 1 m^3^/d wastewater** |
| --- | --- | --- | --- | --- |
| Xianning, Shaanxi | 4/5/2017 | 5 | 15,832.64 | 3166.53 |
| Suzhou, Anhui | 3/27/2017 | 2 | 7,048.73 | 3524.37 |
| Hohhot, Inner Mongolia | 1/5/2017 | 5 | 11,500.00 | 2300.00 |
| Karamay, Sinkiang | 9/29/2016 | 10 | 27,503.96 | 2750.40 |
| Chongqing | 12/15/2016 | 1 | 4,351.00 | 4351.00 |
| Wuhan, Hubei | 1/4/2017 | 8.5 | 3,988.30 | 469.21 |
| Shangluo, Shaanxi | 12/28/2016 | 6 | 38,000.00 | 6333.33 |
| Bozhou, Anhui | 11/17/2016 | 5.5 | 12,000.00 | 2181.82 |
| Jingmen, Hubei | 8/8/2016 | 0.87 | 3,623.97 | 4165.48 |
| Yan’an, Shaanxi | 8/4/2016 | 5 | 8,948.37 | 1789.67 |
| Hefei, Anhui | 6/28/2016 | 10 | 39,500.00 | 3950.00 |
| Changsha, Hunan | 5/23/2016 | 5 | 17,000.00 | 3400.00 |
| Shantou, Guangdong | 1/5/2016 | 7.5 | 18,365.00 | 2448.67 |
| Jinhua, Zhejiang | 12/10/2015 | 3 | 9,182.00 | 3060.67 |
| Rizhao, Shandong | 3/22/2016 | 3.7 | 8,500.00 | 2297.30 |
| Baoding, Hebei | 11/25/2015 | 5 | 10,000.00 | 2000.00 |
| Heze, Shandong | 8/12/2015 | 2 | 10,000.00 | 5000.00 |
| Pingdingshan, Henan | 10/26/2015 | 26 | 86,100.00 | 3311.54 |
| Jinhua, Zhejiang | 11/23/2015 | 13.8 | 28,000.00 | 2028.99 |
| Baoding, Hebei | 8/6/2015 | 1.25 | 1,502.97 | 1202.38 |
| Dalian, Liaoning | 3/13/2014 | 4 | 10,800.00 | 2700.00 |
| Jiujiang, Jiangxi | 1/15/2014 | 4 | 12,000.00 | 3000.00 |
| **Average** |  |  |  | **2974.15** |

*^a^* Effluent quality is all Standard A of first class; all cost has been converted into account for inflation

Annual liquid fertilizer benefit:

Based on the content of nitrogen, phosphorus and potassium, the price of liquid fertilizer is 2000 CNY/m^3^.

SB_1_-SC_4_: $0.158\frac{m^{3}}{d}\times365d\times2000\frac{CNY}{m^{3}}=115340 CNY$

Annual biogas and biowaste benefit:

The price of biogas is 0.75 CNY/m^3^, ranging from 0.5 CNY/m^3^ to 1 CNY/m^3^. The biowaste produced by anaerobic digestion do not have the feasibility of selling because of low content of nutrients.

SC_1_-SC_4_: $0.11\frac{m^{3}}{d}\times365d\times0.75\frac{CNY}{m^{3}}=30 CNY$

The ENPV calculation process:

**Table A.6.** The ENPV calculation process. (×10^4^ CNY)

| SA | Years | t=0 | t=1 | t=2 | t=3 | t=4 | t=5 | t=6 | t=7 | t=8 | t=9 | t=10 |
| --- | --- | --- | --- | --- | --- | --- | --- | --- | --- | --- | --- | --- |
|  | $B_{t}$ | 0 | 0 | 0 | 0 | 0 | 0 | 0 | 0 | 0 | 0 | 0 |
|  | $C_{t}$ | 23.34 | 1.86 | 1.86 | 1.86 | 1.86 | 1.86 | 1.86 | 1.86 | 1.86 | 1.86 | 1.86 |
|  | $\frac{B_{t}-C_{t}}{\left( 1+i \right)^{t}}$ | -23.34 | -1.72 | -1.59 | -1.48 | -1.37 | -1.27 | -1.17 | -1.09 | -1.00 | -0.93 | -0.86 |
|  | Years | t=11 | t=12 | t=13 | t=14 | t=15 | t=16 | t=17 | t=18 | t=19 | t=20 | **ENPV** |
|  | $B_{t}$ | 0 | 0 | 0 | 0 | 0 | 0 | 0 | 0 | 0 | 0 | **-41.60** |
|  | $C_{t}$ | 1.86 | 1.86 | 1.86 | 1.86 | 1.86 | 1.86 | 1.86 | 1.86 | 1.86 | 1.86 |  |
|  | $\frac{B_{t}-C_{t}}{\left( 1+i \right)^{t}}$ | -0.80 | -0.74 | -0.68 | -0.63 | -0.59 | -0.54 | -0.50 | -0.47 | -0.43 | -0.40 |  |
| SB_1_ | Years | t=0 | t=1 | t=2 | t=3 | t=4 | t=5 | t=6 | t=7 | t=8 | t=9 | t=10 |
|  | $B_{t}$ | 0 | 11.53 | 11.53 | 11.53 | 11.53 | 11.53 | 11.53 | 11.53 | 11.53 | 11.53 | 11.53 |
|  | $C_{t}$ | 62.53 | 5.13 | 5.13 | 5.13 | 5.13 | 5.13 | 5.13 | 5.13 | 5.13 | 5.13 | 5.13 |
|  | $\frac{B_{t}-C_{t}}{\left( 1+i \right)^{t}}$ | -62.53 | 5.93 | 5.49 | 5.08 | 4.70 | 4.36 | 4.04 | 3.73 | 3.46 | 3.20 | 2.97 |
|  | Years | t=11 | t=12 | t=13 | t=14 | t=15 | t=16 | t=17 | t=18 | t=19 | t=20 | **ENPV** |
|  | $B_{t}$ | 11.53 | 11.53 | 11.53 | 11.53 | 11.53 | 11.53 | 11.53 | 11.53 | 11.53 | 11.53 | **0.35** |
|  | $C_{t}$ | 5.13 | 5.13 | 5.13 | 5.13 | 5.13 | 5.13 | 5.13 | 5.13 | 5.13 | 5.13 |  |
|  | $\frac{B_{t}-C_{t}}{\left( 1+i \right)^{t}}$ | 2.75 | 2.54 | 2.35 | 2.18 | 2.02 | 1.87 | 1.73 | 1.60 | 1.48 | 1.37 |  |
| SB_2_ | Years | t=0 | t=1 | t=2 | t=3 | t=4 | t=5 | t=6 | t=7 | t=8 | t=9 | t=10 |
|  | $B_{t}$ | 0 | 11.53 | 11.53 | 11.53 | 11.53 | 11.53 | 11.53 | 11.53 | 11.53 | 11.53 | 11.53 |
|  | $C_{t}$ | 61.53 | 5.47 | 5.47 | 5.47 | 5.47 | 5.47 | 5.47 | 5.47 | 5.47 | 5.47 | 5.47 |
|  | $\frac{B_{t}-C_{t}}{\left( 1+i \right)^{t}}$ | -61.53 | 5.61 | 5.20 | 4.81 | 4.46 | 4.13 | 3.82 | 3.54 | 3.28 | 3.03 | 2.81 |
|  | Years | t=11 | t=12 | t=13 | t=14 | t=15 | t=16 | t=17 | t=18 | t=19 | t=20 | **ENPV** |
|  | $B_{t}$ | 11.53 | 11.53 | 11.53 | 11.53 | 11.53 | 11.53 | 11.53 | 11.53 | 11.53 | 11.53 | **-1.99** |
|  | $C_{t}$ | 5.47 | 5.47 | 5.47 | 5.47 | 5.47 | 5.47 | 5.47 | 5.47 | 5.47 | 5.47 |  |
|  | $\frac{B_{t}-C_{t}}{\left( 1+i \right)^{t}}$ | 2.60 | 2.41 | 2.23 | 2.06 | 1.91 | 1.77 | 1.64 | 1.52 | 1.41 | 1.30 |  |
| SC_1_ | Years | t=0 | t=1 | t=2 | t=3 | t=4 | t=5 | t=6 | t=7 | t=8 | t=9 | t=10 |
|  | $B_{t}$ | 0 | 11.53 | 11.53 | 11.53 | 11.53 | 11.53 | 11.53 | 11.53 | 11.53 | 11.53 | 11.53 |
|  | $C_{t}$ | 68.92 | 5.43 | 5.43 | 5.43 | 5.43 | 5.43 | 5.43 | 5.43 | 5.43 | 5.43 | 5.43 |
|  | $\frac{B_{t}-C_{t}}{\left( 1+i \right)^{t}}$ | -68.92 | 5.65 | 5.24 | 4.85 | 4.49 | 4.16 | 3.85 | 3.56 | 3.30 | 3.06 | 2.83 |
|  | Years | t=11 | t=12 | t=13 | t=14 | t=15 | t=16 | t=17 | t=18 | t=19 | t=20 | **ENPV** |
|  | $B_{t}$ | 11.53 | 11.53 | 11.53 | 11.53 | 11.53 | 11.53 | 11.53 | 11.53 | 11.53 | 11.53 | **-8.96** |
|  | $C_{t}$ | 5.43 | 5.43 | 5.43 | 5.43 | 5.43 | 5.43 | 5.43 | 5.43 | 5.43 | 5.43 |  |
|  | $\frac{B_{t}-C_{t}}{\left( 1+i \right)^{t}}$ | 2.62 | 2.43 | 2.25 | 2.08 | 1.93 | 1.78 | 1.65 | 1.53 | 1.42 | 1.31 |  |
| SC_2_ | Years | t=0 | t=1 | t=2 | t=3 | t=4 | t=5 | t=6 | t=7 | t=8 | t=9 | t=10 |
|  | $B_{t}$ | 0 | 11.53 | 11.53 | 11.53 | 11.53 | 11.53 | 11.53 | 11.53 | 11.53 | 11.53 | 11.53 |
|  | $C_{t}$ | 66.92 | 5.68 | 5.68 | 5.68 | 5.68 | 5.68 | 5.68 | 5.68 | 5.68 | 5.68 | 5.68 |
|  | $\frac{B_{t}-C_{t}}{\left( 1+i \right)^{t}}$ | -66.92 | 5.42 | 5.02 | 4.65 | 4.31 | 3.99 | 3.69 | 3.42 | 3.16 | 2.93 | 2.71 |
|  | Years | t=11 | t=12 | t=13 | t=14 | t=15 | t=16 | t=17 | t=18 | t=19 | t=20 | **ENPV** |
|  | $B_{t}$ | 11.53 | 11.53 | 11.53 | 11.53 | 11.53 | 11.53 | 11.53 | 11.53 | 11.53 | 11.53 | **-9.42** |
|  | $C_{t}$ | 5.68 | 5.68 | 5.68 | 5.68 | 5.68 | 5.68 | 5.68 | 5.68 | 5.68 | 5.68 |  |
|  | $\frac{B_{t}-C_{t}}{\left( 1+i \right)^{t}}$ | 2.51 | 2.33 | 2.15 | 1.99 | 1.85 | 1.71 | 1.58 | 1.47 | 1.36 | 1.26 |  |
| SC_3_ | Years | t=0 | t=1 | t=2 | t=3 | t=4 | t=5 | t=6 | t=7 | t=8 | t=9 | t=10 |
|  | $B_{t}$ | 0 | 11.53 | 11.53 | 11.53 | 11.53 | 11.53 | 11.53 | 11.53 | 11.53 | 11.53 | 11.53 |
|  | $C_{t}$ | 71.41 | 6.08 | 6.08 | 6.08 | 6.08 | 6.08 | 6.08 | 6.08 | 6.08 | 6.08 | 6.08 |
|  | $\frac{B_{t}-C_{t}}{\left( 1+i \right)^{t}}$ | -71.41 | 5.05 | 4.68 | 4.33 | 4.01 | 3.71 | 3.44 | 3.18 | 2.95 | 2.73 | 2.53 |
|  | Years | t=11 | t=12 | t=13 | t=14 | t=15 | t=16 | t=17 | t=18 | t=19 | t=20 | **ENPV** |
|  | $B_{t}$ | 11.53 | 11.53 | 11.53 | 11.53 | 11.53 | 11.53 | 11.53 | 11.53 | 11.53 | 11.53 | **-17.87** |
|  | $C_{t}$ | 6.08 | 6.08 | 6.08 | 6.08 | 6.08 | 6.08 | 6.08 | 6.08 | 6.08 | 6.08 |  |
|  | $\frac{B_{t}-C_{t}}{\left( 1+i \right)^{t}}$ | 2.34 | 2.17 | 2.01 | 1.86 | 1.72 | 1.59 | 1.47 | 1.36 | 1.26 | 1.17 |  |
| SC_4_ | Years | t=0 | t=1 | t=2 | t=3 | t=4 | t=5 | t=6 | t=7 | t=8 | t=9 | t=10 |
|  | $B_{t}$ | 0 | 11.53 | 11.53 | 11.53 | 11.53 | 11.53 | 11.53 | 11.53 | 11.53 | 11.53 | 11.53 |
|  | $C_{t}$ | 69.41 | 6.70 | 6.70 | 6.70 | 6.70 | 6.70 | 6.70 | 6.70 | 6.70 | 6.70 | 6.70 |
|  | $\frac{B_{t}-C_{t}}{\left( 1+i \right)^{t}}$ | -69.41 | 4.48 | 4.14 | 3.84 | 3.55 | 3.29 | 3.05 | 2.82 | 2.61 | 2.42 | 2.24 |
|  | Years | t=11 | t=12 | t=13 | t=14 | t=15 | t=16 | t=17 | t=18 | t=19 | t=20 | **ENPV** |
|  | $B_{t}$ | 11.53 | 11.53 | 11.53 | 11.53 | 11.53 | 11.53 | 11.53 | 11.53 | 11.53 | 11.53 | **-21.96** |
|  | $C_{t}$ | 6.70 | 6.70 | 6.70 | 6.70 | 6.70 | 6.70 | 6.70 | 6.70 | 6.70 | 6.70 |  |
|  | $\frac{B_{t}-C_{t}}{\left( 1+i \right)^{t}}$ | 2.07 | 1.92 | 1.78 | 1.65 | 1.52 | 1.41 | 1.31 | 1.21 | 1.12 | 1.04 |  |

| *System for urban area* |
| --- |
| 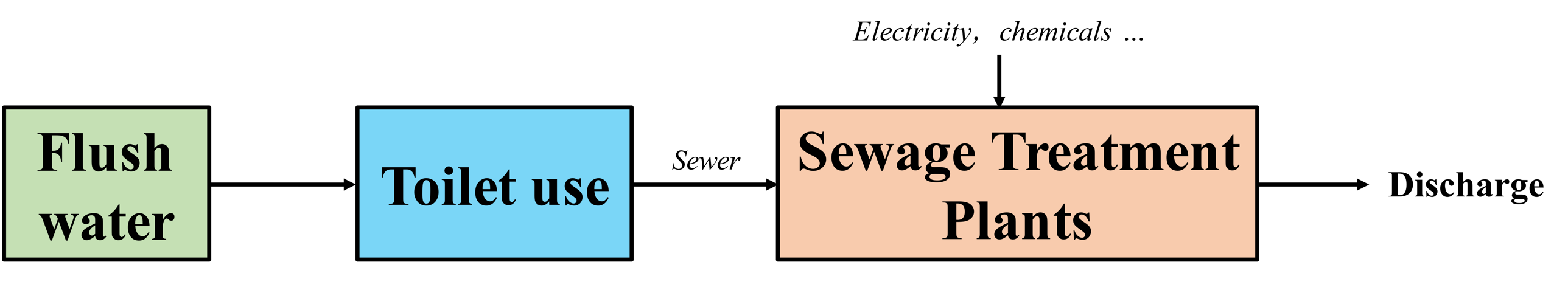 |
| *System for rural area* |
|  |

**Fig. A.1.** Simplified schematic of Scenario A





**Fig.A.2.** Simplified schematic of Scenario B


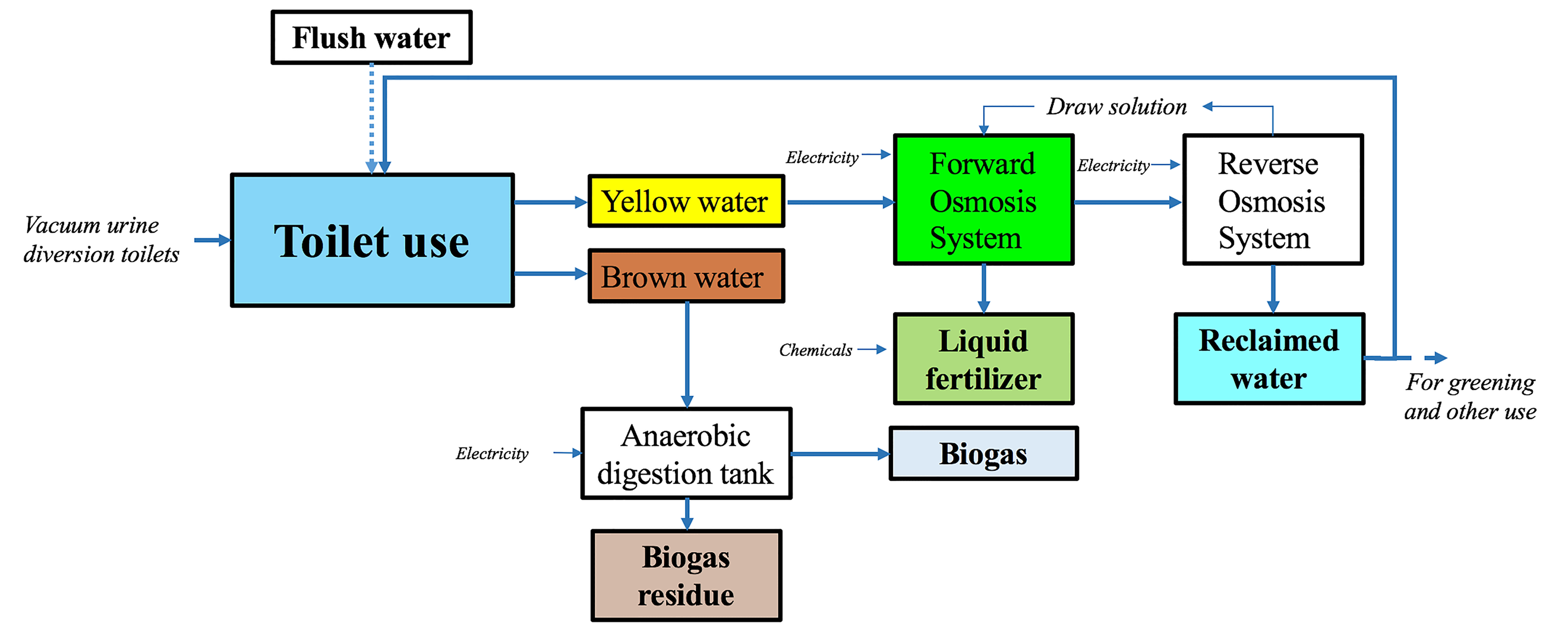


**Fig.A.3.** Simplified schematic of Scenario C
